# Transient Recombinant Protein Production in Glycoengineered *Nicotiana benthamiana* Cell Suspension Culture

**DOI:** 10.3390/ijms19041205

**Published:** 2018-04-16

**Authors:** Sara C. Sukenik, Kalimuthu Karuppanan, Qiongyu Li, Carlito B. Lebrilla, Somen Nandi, Karen A. McDonald

**Affiliations:** 1 Department of Chemical Engineering, University of California, Davis, CA 95616, USA; scsukenik@ucdavis.edu (S.C.S.); kkaruppanan@ucdavis.edu (K.K.); snandi@ucdavis.edu (S.N.); 2 Department of Chemistry, University of California, Davis, CA 95616, USA; qyuli@ucdavis.edu (Q.L.); cblebrilla@ucdavis.edu (C.B.L.); 3 Department of Biochemistry and Molecular Medicine, University of California, Davis, CA 95616, USA; 4 Foods for Health Institute, University of California, Davis, CA 95616, USA; 5 Global HealthShare Initiative, University of California, Davis, CA 95616, USA

**Keywords:** *Agrobacterium tumefaciens*, plant cell culture, co-culture, recombinant protein expression, N-glycosylation

## Abstract

Transient recombinant protein production is a promising alternative to stable transgenic systems, particularly for emergency situations in which rapid production of novel therapeutics is needed. In plants, *Agrobacterium tumefaciens* can be used as a gene delivery vector for transient expression. A potential barrier for plant-based production of human therapeutics is that different glycosylation patterns are found on plant and mammalian proteins. Since glycosylation can affect the efficacy, safety and stability of a therapeutic protein, methods to control glycan structures and distributions in plant-based systems would be beneficial. In these studies, we performed *Agrobacterium*-mediated transient expression in glycoengineered plant cell suspension cultures. To reduce the presence of plant-specific glycans on the product, we generated and characterized cell suspension cultures from β-1,2-xylosyltransferase and α-1,3-fucosyltransferase knockdown *Nicotiana benthamiana*. An anthrax decoy fusion protein was transiently produced in these glycoengineered plant cell suspension cultures through co-culture with genetically engineered *Agrobacterium*. The mass ratio of *Agrobacterium* to plant cells used was shown to impact recombinant protein expression levels. N-glycosylation analysis on the anthrax decoy fusion protein produced in glycoengineered *N. benthamiana* showed a dramatic reduction in plant-specific N-glycans. Overall, the results presented here demonstrate the feasibility of a simple, rapid and scalable process for transient production of recombinant proteins without plant-specific glycans in a glycoengineered plant cell culture host.

## 1. Introduction

Rapid, large-scale production of novel drugs or vaccines would be invaluable in emergency situations, such as an infectious disease outbreak or bioterrorist attack. Since developing a stable transgenic cell line can take months to years, transient expression systems may be better suited for these situations. *Agrobacterium tumefaciens*, a plant pathogen that can be genetically engineered to eliminate virulence genes and instead deliver a gene of interest, has been widely used for transient expression in whole plants through a process known as agroinfiltration [1]. Given that the required *Agrobacterium* constructs can be produced in as little as two weeks, this system can be utilized for rapid response applications. In response to the 2009 A/H1N1 outbreak, the first purified lot of research grade influenza vaccine was produced using agroinfiltration in plants within 3 weeks of sequence availability [2].

While agroinfiltration can produce recombinant proteins with short lead times, limitations of a whole plant transient expression system include the need to grow and manipulate whole plants and challenges associated with purification of the product from plant biomass. We are investigating *Agrobacterium-*mediated transient expression in plant cell suspension cultures as an alternative production system. A transient cell culture system would have similarly short lead times yet could allow controlled and optimized environmental conditions in a bioreactor for plant growth and protein production while simplifying purification by targeting the protein for secretion. Additionally, manufacturing capacity for agroinfiltration is currently limited, while a transient plant cell culture system could utilize existing cell culture manufacturing infrastructure. Transient plant-made pharmaceutical production has been demonstrated at commercial-scale [3]. However, only a few facilities of this type exist, including those operated by Kentucky BioProcessing (Owensboro, KY, USA), Medicago (Durham, NC, USA and Quebec City, QC, Canada), iBio (Bryan, TX, USA), Icon Genetics (Halle, Germany) and Fraunhofer CMB (Newark, DE, USA) [4,5,6,7,8]. Recently, plant cell packs have also been used for transient expression [9]. In this system, a porous cell pack is created using vacuum filtration to remove the culture media. *Agrobacterium* is added to the cell pack for a brief incubation period, then removed by vacuum filtration. Secreted proteins can later be harvested by flowing a solution through the cell pack. However, similar to whole plant processes, a cell pack process would also require specialized commercial manufacturing facilities.

Plant cell culture affords several advantages for recombinant protein production including capacity for post-translational modifications, low risk of viral contamination and inexpensive culture media [10]. Currently, there is one FDA approved plant made pharmaceutical, Elelyso^®^, a recombinant human glucocerebrosidase treatment for Gaucher’s disease, produced in stable transgenic carrot cell culture by Protalix Biotherapeutics [11]. A potential limitation of plant-based recombinant protein production is that different glycosylation patterns are present on mammalian and plant proteins [12]. Although the plant glycans on Elelyso^®^ do not affect the product’s safety or efficacy [11], glycoengineering of plant produced proteins is being explored to enhance their function and stability [13,14]. To remove plant-specific β(1,2)-xylosylation and core α(1,3)-fucosylation, RNA interference was used to knockdown the β-1,2-xylosyltransferase and α-1,3-fucosyltransferase genes in *Nicotiana benthamiana* [15]. In mouse studies, antibodies against Ebola produced in these glycoengineered *N. benthamiana* plants had enhanced efficacy compared to antibodies made in mammalian cell culture [16]. In subsequent studies, an optimized combination of plant made monoclonal antibodies (ZMapp) rescued 100% of rhesus macaques from a lethal Ebola virus challenge [17].

In this study, we generated and characterized cell suspension cultures from β-1,2-xylosyltransferase and α-1,3-fucosyltransferase knockdown *N. benthamiana* (ΔXTFT *N. benthamiana*). To test *Agrobacterium-*mediated transient recombinant protein expression in these glycoengineered cell suspension cultures, we produced an anthrax decoy-Fc fusion protein (CMG2-Fc). CMG2-Fc consists of a portion of the human capillary morphogenesis gene 2 (CMG2) protein, a receptor for the anthrax protective antigen toxin [18,19], fused to human IgG Fc through a linker. CMG2-Fc has previously been produced transiently in whole *N. benthamiana* plants and has been shown to protect rabbits against a lethal inhalational anthrax challenge [20,21]. Finally, we analyzed the N-glycosylation patterns on CMG2-Fc produced transiently in ΔXTFT *N. benthamiana* cell suspension cultures. To the best of our knowledge, this is the first report of glycoengineering in a transient plant cell suspension culture system.

## 2. Results

### 2.1. Generation of ΔXTFT N. benthamiana Cell Suspension Cultures

**Figure 1:**
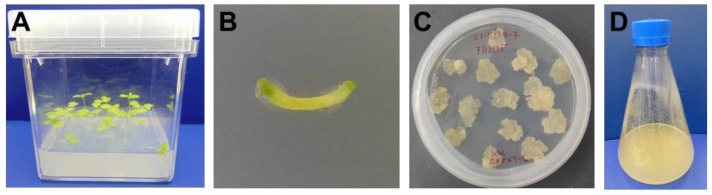
Callus generation. (**A**) Aseptically grown ΔXTFT *N. benthamiana* plants; (**B**) Explant from ΔXTFT *N. benthamiana* plant on semi-solid media; (**C**) ΔXTFT *N. benthamiana* callus formed from dedifferentiation of explants and grown on semi-solid media; (**D**) ΔXTFT *N. benthamiana* cells in suspension culture.

**Figure 2:**
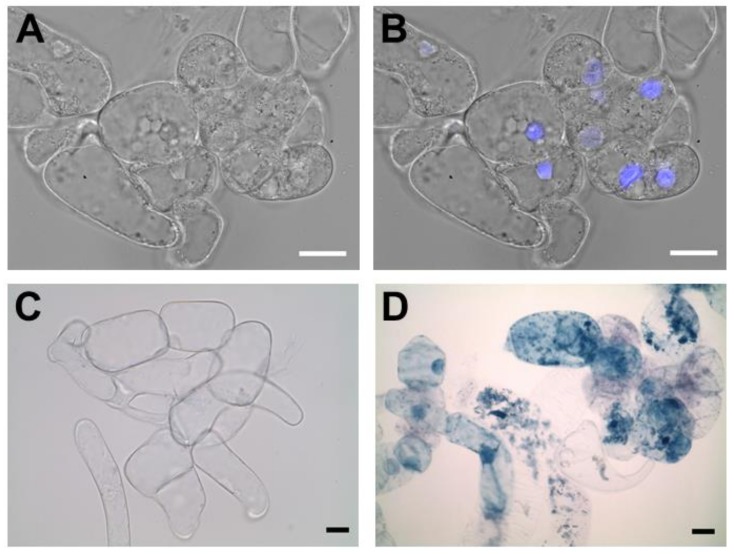
Morphology of ΔXTFT *N. benthamiana* cell suspension culture. Scale bars indicate 20 µm. (**A**,**B**) Same field of view; (**B**) Nuclear staining with DAPI; (**C**) Cells stained with Evans blue; (**D**) Cells treated with 70% ethanol for 3 h prior to Evans blue staining.

The ΔXTFT *N. benthamiana* callus line was generated from aseptically grown plants (Figure 1A) by culturing explants on semi-solid media (Figure 1B). After dedifferentiation of the plant tissue, calli were maintained on semi-solid media by subculturing monthly (Figure 1C). Suspension cultures were generated by sieving calli through a 280 μm mesh screen into liquid culture media (Figure 1D). Microscopic observations of nuclear stained ΔXTFT *N. benthamiana* cell culture showed multicellular aggregates (Figure 2A,B). Staining with Evans blue indicated that most cells in suspension culture were viable (Figure 2C), while positive control cells treated with ethanol were nonviable (Figure 2D).

### 2.2. Characterization of ΔXTFT N. benthamiana Cell Suspension Cultures

**Figure 3:**
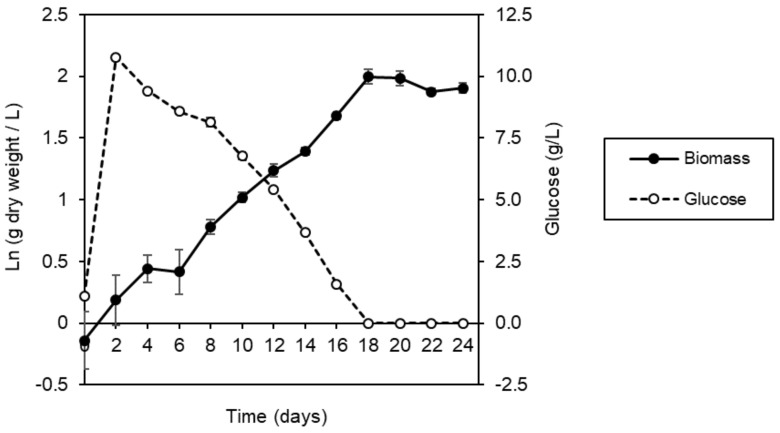
Growth of ΔXTFT *N. benthamiana* cell suspension culture in a 1 L shake flask with 200 mL culture volume. The average value and standard deviation from triplicate samples are shown.

The growth kinetics of ΔXTFT *N. benthamiana* cell suspension cultures were determined using 200 mL of culture in a 1 L shake flask, which was incubated at 28 °C and 140 rpm in a medium containing Murashige and Skoog basal salts. Within the first few days, the sucrose provided in the culture media was hydrolyzed to glucose and fructose. By day 2 after subculturing, sucrose was depleted to less than 1 g/L. Figure 3 shows the natural logarithm of the dry cell biomass concentration and the media glucose concentration. When the natural logarithm is plotted, a linear increase in biomass concentration indicates exponential growth and is observed from day 0. By day 18, glucose was fully consumed and the dry cell biomass concentration began to decrease. Growth kinetic parameters are summarized in Table 1. The maximum specific growth rate was 0.113 day^−1^. This value is lower than previously found for wild type *N. benthamiana* cell suspension cultures (0.26 day^−1^) [22]. However, the wild type growth rate was determined in a bioreactor under controlled dissolved oxygen (40% air saturation) and a different temperature (25 °C) compared with the shake flask cultures; these differences could result in different growth kinetics.

### 2.3. Effect of Varying Agrobacterium Amount and Media Volume During Co-Culture

**Figure 4:**
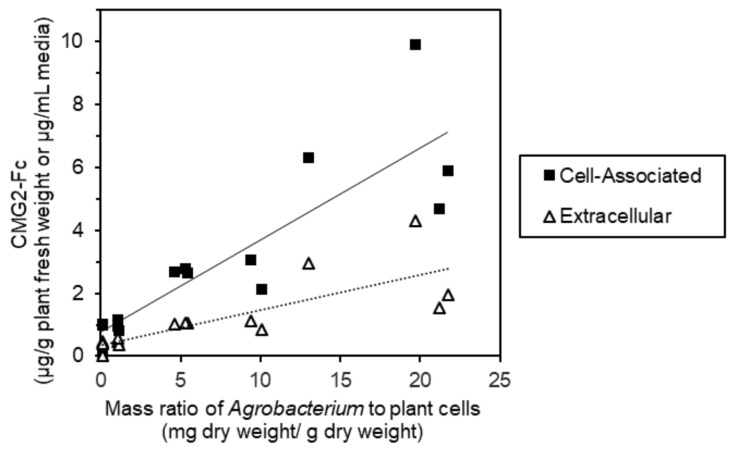
CMG2-Fc expression after 7 days of co-culture quantified by an enzyme-linked immunosorbent assay (ELISA) for varying *Agrobacterium* to *N. benthamiana* mass ratios. Co-culture was performed in 3 separate flasks for each mass ratio target level. Linear regression trendlines are shown for both the cell-associated (solid line) and extracellular (dotted line) samples.

Various amounts of *Agrobacterium* were added to plant cells to assess the effect of this parameter on CMG2-Fc expression levels. *Agrobacterium* was resuspended to varying OD_600_, such that equal volumes (1.5 mL) with different masses of *Agrobacterium* could be added to each flask (containing 48 mL of cell suspension culture). Since the *Agrobacterium* vector used included a SEKDEL sequence on the CMG2-Fc protein, CMG2-Fc was expected to be retained in the endoplasmic reticulum. However, CMG2-Fc was detected in both the culture media and biomass extract after 7 days of co-culture. As the mass ratio of *Agrobacterium* increased from approximately 0.1 to 20 mg dry weight of *Agrobacterium/*g dry weight of plant cells, both cell-associated and extracellular CMG2-Fc production increased (Figure 4). At the time of *Agrobacterium* addition, the average plant cell dry weight of the flasks was 6.4 g/L with a standard deviation of 0.6 g/L. In a mock addition control, the same volume of *Agrobacterium* resuspension buffer (10 mM MgCl_2_, 20 mM MES and 150 μM acetosyringone) was added to plant cell cultures. To ensure that production of CMG2-Fc detected was not from the *Agrobacterium* itself, an equivalent amount of *Agrobacterium* as used in the 20 mg/g mass ratio target was added to plant cell culture media and incubated under the same conditions. No CMG2-Fc was detected using an enzyme-linked immunosorbent assay (ELISA) for either control.

**Figure 5:**
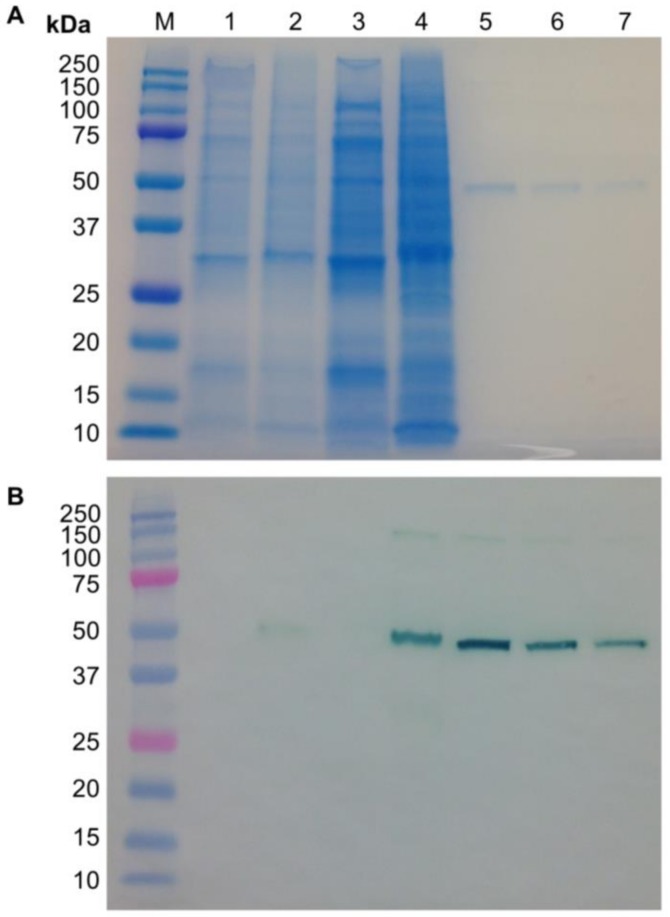
(**A**) Sodium dodecyl sulfate polyacrylamide gel electrophoresis (SDS-PAGE) and (**B**) immunoblot analysis of crude CMG2-Fc cell-associated samples. Lane **M**, molecular weight marker; lane **1**, mock addition crude extract; lane **2**, co-culture crude extract; lane **3**, five-fold concentrated mock addition crude extract; lane **4**, five-fold concentrated co-culture crude extract; lane **5**, 250 ng CMG2-Fc standard; lane **6**, 150 ng CMG2-Fc standard; lane **7**, 100 ng CMG2-Fc standard.

Sodium dodecyl sulfate polyacrylamide gel electrophoresis (SDS-PAGE) and immunoblot analysis were performed using cell-associated samples from the highest expressing co-culture flask and the mock addition control (Figure 5). In addition to the crude extracts, samples that had been concentrated five-fold were analyzed. No bands were observed on the immunoblot for the mock addition control samples (lanes 1 and 3). A faint band was observed in the crude co-culture extract at the expected size (lane 2). After concentrating the co-culture sample, a stronger band was observed at the expected size (lane 4). A less distinct band in the concentrated co-culture sample was observed at a higher molecular weight. A band at this higher size also appears in the CMG2-Fc protein standard (lanes 5–7) and may represent protein aggregation.

**Figure 6:**
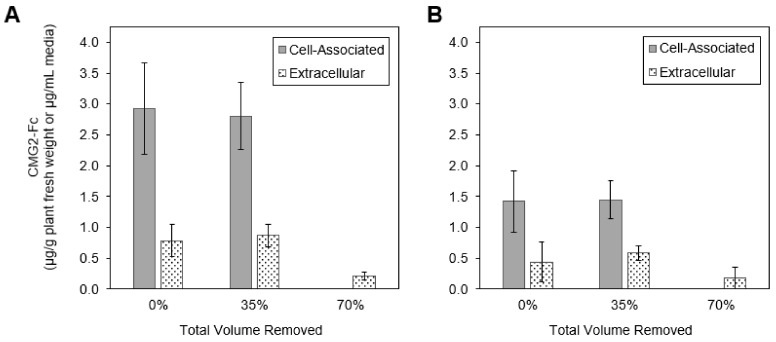
CMG2-Fc expression quantified by ELISA after media reduction during co-culture for two mass ratios of *Agrobacterium* to plant cells: (**A**) 11.5 ± 1.5 (average ± standard deviation) mg dry weight *Agrobacterium/*g dry weight plant cell and (**B**) 23.2 ± 3.5 mg dry weight *Agrobacterium/*g dry weight plant cell. Error bars represent the standard deviation from triplicate flasks.

The total co-culture volume was varied to assess the impact of simultaneously increasing the concentrations of *Agrobacterium* and plant cells (Figure 6). To reduce the total volume while maintaining the same total biomass, the plant cell culture (48 mL) was centrifuged and a portion of the supernatant was removed. The same volume of *Agrobacterium* (1.1 mL) was added to each flask after media removal. Removing 35% of the culture volume did not affect CMG2-Fc expression levels. However, when 70% of the culture volume was removed, cell-associated CMG2-Fc was not detected and extracellular CMG2-Fc decreased. The same responses were observed for the two mass ratio levels of *Agrobacterium* to plant cells that were tested. In this experiment, higher expression was observed at an average mass ratio of 11.5 mg dry weight *Agrobacterium*/g dry weight plant cell than at 23.2 mg/g. Prior to media removal, the average plant cell dry weight of the flasks was 2.7 g/L with a standard deviation of 0.4 g/L. Again, extracellular CMG2-Fc was detected although a SEKDEL sequence was included on the CMG2-Fc protein.

### 2.4. Analysis of N-Glycan Distribution on CMG2-Fc

**Figure 7:**
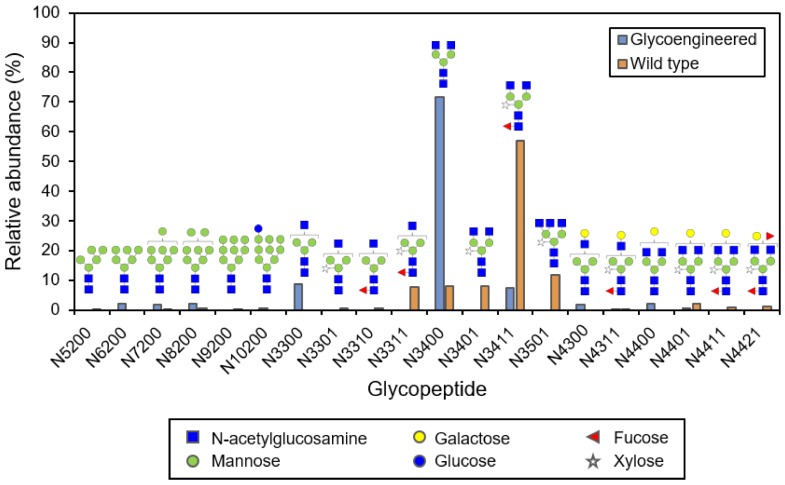
Distribution of N-glycans on CMG2-Fc produced transiently in ΔXTFT *N. benthamiana* cell culture (glycoengineered) and through agroinfiltration of wild type *N. benthamiana* plants (wild type). Labels on the *x*-axis represent the number of monosaccharides in N-linked glycans in the following order: hexose (mannose, glucose and galactose), N-acetylglucosamine, fucose and xylose.

CMG2-Fc contains only one N-glycosylation site, which is located on the Fc portion. After purification of cell-associated CMG2-Fc using Protein A chromatography, the N-glycan distribution was analyzed (Figure 7). In this experiment, an *Agrobacterium* vector that targeted CMG2-Fc for secretion was used to allow more complex glycoforms. Similar cell-associated and extracellular CMG2-Fc levels for the ER retained and extracellularly-targeted variants have been observed in co-culture experiments. Due to the lower concentration of extracellular CMG2-Fc (due to dilution in the medium) and ease of concentration of the cell-associated CMG2-Fc using centrifugation, we isolated the CMG2-Fc product from the cell aggregates. The most abundant glycan structure (72%) identified on cell-associated CMG2-Fc produced in ΔXTFT *N. benthamiana* was a complex N-glycan but lacked β(1,2)-xylose and α(1,3)-linked core fucose. In contrast, the most abundant structure (57%) on CMG2-Fc produced previously by our group through agroinfiltration with the same *Agrobacterium* vector in wild type *N. benthamiana* plants included β(1,2)-xylose and α(1,3)-linked core fucose [20]. This structure containing β(1,2)-xylose and α(1,3)-linked core fucose was found at a much lower abundance (8%) on CMG2-Fc produced in glycoengineered cell cultures. The high level of complex glycoforms with one or two N-acetylglucosamines indicates that the cell-associated CMG2-Fc was likely outside of the cell membrane. Altogether, these results demonstrate the ability to produce a recombinant protein with complex glycoforms and very low levels of plant-specific glycans using the β-1,2-xylosyltransferase and α-1,3-fucosyltransferase knockdown cell line in the suspension co-culture process.

## 3. Discussion and Future Work

While the data presented here demonstrate the feasibility of glycoengineering in a transient cell culture system, additional process development is needed. In shake flask studies, we observed that the optimal mass ratio of *Agrobacterium* to plant cells varied from experiment to experiment (Figure 4 and Figure 6). Although 7-day old plant cell cultures were used in both experiments, the average starting dry biomass concentrations of the plant cells differed (6.4 ± 0.6 g/L and 2.7 ± 0.4 g/L), possibly due to varied lag phase durations. Given this observed variation, it is hypothesized that the plant cell growth phase impacts the expression level obtained for any given mass ratio of *Agrobacterium* added. The tighter environmental control that can be achieved in a bioreactor compared to a shake flask would improve consistency of cell growth from batch to batch. In a single run, higher expression levels of a reporter gene in co-culture were obtained in a bioreactor compared to simultaneous shake flask cultures [23]. However, the reproducibility of co-culture expression levels through multiple bioreactor runs has not yet been reported. In addition to bioreactor-based plant cell growth and co-culture, additional experiments are needed to optimize *Agrobacterium* growth and virulence induction for this transient cell culture expression system.

Previously reported expression levels for recombinant proteins produced in stable transgenic plant cell suspension cultures range from several hundred μg/L to several hundred mg/L [24]. The expression levels reported here for transient expression in plant cell suspension cultures are within that range. Transient expression in mammalian cell cultures is commonly used for biologic drug development and screening, however product titers at scales greater than 1 liter range from less than 1 to 80 mg/L [25,26]. Compared to transient expression in mammalian cell culture, our system also has greater potential for economical large-scale production. The plant co-culture system uses a self-replicating vector that is added into the culture media, eliminating the need for large quantities of purified plasmid DNA and expensive transfection reagents.

CMG2-Fc expression levels resulting from agroinfiltration of whole plants were two orders of magnitude higher than the expression levels obtained in our transient cell culture studies [20]. Further optimization of the *Agrobacterium*-plant cell interaction in the cell culture system is needed to enhance recombinant protein production. Previously, auxotrophic strains have been developed to control the growth of *Agrobacterium* during co-culture and were shown to increase production of a reporter protein [27]. Cell cycle synchronization, achieved by sucrose depletion followed by nutrient addition, is another strategy that has been shown to increase reporter gene expression [23]. Other co-culture studies have demonstrated varied expression levels in different *Nicotiana* species and have used viral vector systems to enhance expression [28,29,30]. Viral gene silencing suppressors have also been shown to enhance expression levels in co-culture systems, though to a lesser extent than in whole plant agroinfiltration [29,31]. Our results demonstrate that the effects of process parameters, such as the mass ratio of *Agrobacterium* added, also need to be considered during optimization of this production platform.

In our studies, CMG2-Fc was detected in the culture media, even when an *Agrobacterium* construct designed to retain the protein in the endoplasmic reticulum was used. Co-culture with *Agrobacterium* under these conditions may lead to plant cell lysis, resulting in detectable levels of extracellular CMG2-Fc. The levels of extracellular product reported here are three orders of magnitude higher than Boivin et al. previously reported for an antibody produced by co-culturing *Agrobacterium* and *N. benthamiana* in suspension cultures [31].

Finally, the results presented here demonstrate that a transgenic plant cell line can be used to affect N-glycosylation on proteins expressed transiently in cell culture. N-glycans on recombinant proteins produced in plant cell suspension cultures typically contain β(1,2)-xylose and α(1,3)-linked core fucose, as recently shown for recombinant butyrylcholinesterase produced in stable transgenic rice cell cultures [32]. In contrast, the most abundant glycoform identified in our study lacked these plant-specific residues and is the same as previously reported for a human antibody produced through agroinfiltration in ΔXTFT *N. benthamiana* plants [15]. Although our predominant structure lacked β(1,2)-xylose and α(1,3)-linked core fucose, structures with these plant-specific residues were also detected at lower levels on CMG2-Fc. Recently, CRISPR/Cas9 genome editing has been used to knockout the β-1,2-xylosyltransferase and α-1,3-fucosyltransferase genes in *Nicotiana tabacum* BY-2 cells. After further transforming these knockout cell lines with a gene of interest, the resulting recombinant protein lacked β(1,2)-xylose and α(1,3)-fucose [33,34]. The co-culture method presented here could utilize similar knockout lines to completely remove plant-specific glycosylation patterns in a transient cell culture system. Stable transformation of the mammalian sialylation pathway into ΔXTFT *N. benthamiana* plants has also been demonstrated [35]. Cell suspension cultures generated from these transgenic plants could be used in our co-culture system to produce sialylated proteins. Alternatively, in vitro enzymatic modification could be performed following production and purification to further engineer the N-glycans produced using this system.

## 4. Materials and Methods

### 4.1. ΔXTFT N. benthamiana Callus Generation and Maintenance

ΔXTFT *N. benthamiana* seeds were sterilized by rinsing with sterile distilled water, submerging in 75% ethanol for 30 s and submerging in 1% sodium hypochlorite for 5 min. After rinsing with sterile distilled water, seeds were transferred to tissue culture boxes containing plant growth media (2.165 g/L Murashige and Skoog basal salt mixture [36] (PhytoTechnology Laboratories, Shawnee Mission, KS, USA), 10 g/L sucrose and 8 g/L Phytagar (Thermo Fisher Scientific, Waltham, MA, USA)). Boxes were sealed and incubated at room temperature in a dark box for 4 days, then moved to incubation at room temperature with a controlled light and dark cycle.

After 8 weeks, a scalpel was used to cut explants from the ΔXTFT *N. benthamiana* plants, which were placed onto callus generation plates (30 g/L sucrose, 4.3 g/L Murashige and Skoog basal salt mixture [36], 0.1 g/L myo-inositol, 0.204 g/L potassium phosphate monobasic, 0.5 mg/L nicotinic acid, 0.5 mg/L thiamine hydrochloride, 0.5 mg/L pyridoxine hydrochloride, 0.4 mg/L 2,4-dichlorophenoxyacetic acid, 0.1 mg/L kinetin and 7.5 g/L Phytagar). After 3 weeks, calli were transferred to new callus generation plates. Following this initial callus generation, plates with 0.2 mg/L 2,4-dichlorophenoxyacetic acid were used. As a gelling agent, plates contained either 7.5 g/L of Phytagar or 1.8 g/L of Gelzan (Sigma-Aldrich, St. Louis, MO, USA). To maintain callus, small portions of calli were transferred to new plates monthly.

### 4.2. Preparation and Maintenance of ΔXTFT N. benthamiana Cell Suspension Cultures

ΔXTFT *N. benthamiana* cell suspension cultures were prepared by pressing callus through a 280 μm mesh screen and combining with suspension culture media (30 g/L sucrose, 4.3 g/L Murashige and Skoog basal salt mixture [36], 0.1 g/L myo-inositol, 0.204 g/L potassium phosphate monobasic, 10 mg/L nicotinic acid, 10 mg/L thiamine hydrochloride, 5 mg/L pyridoxine hydrochloride, 2 mg/L 2,4-dichlorophenoxyacetic acid and 0.1 mg/L kinetin). Suspension cultures were incubated in a benchtop shaker at 140 rpm and 28 °C in the dark and maintained by periodically transferring an inoculum to new shake flasks with fresh media. The working volume used for shake flasks was 20% of the total flask volume.

### 4.3. Characterization of ΔXTFT N. benthamiana Cell Suspension Cultures

The viability of ΔXTFT *N. benthamiana* cell suspension culture was determined using Evans blue staining. Two μL of Evans blue stain solution (1% *w*/*v*) were added to 200 μL of ΔXTFT *N. benthamiana* suspension culture and incubated for 10 min at 25 °C. As a positive control, ΔXTFT *N. benthamiana* cells were treated with 70% ethanol for 3 h prior to Evans blue staining. Unbound Evans blue stain was removed by washing three times with sterile deionized water prior to microscopic observation. Nucleic acid staining was performed using the fluorescent dye DAPI (4′,6-diamidino-2-phenylindole). Twenty μL of DAPI stain (50% glycerol, 1% phosphate-buffered saline (PBS) and 1 µg/mL DAPI) were added to 200 μL of ΔXTFT *N. benthamiana* suspension culture and incubated for 5 min at 25 °C. Unbound DAPI stain was removed by washing three times with sterile PBS buffer prior to microscopic observation. Fluorescent cell images were captured using a Nikon Eclipse (E400) microscope (Nikon, Melville, NY, USA). Cell viability was observed under brightfield illumination using a Nikon Optiphot-2 (Nikon, Melville, NY, USA). Nucleic acid staining was observed in fluorescence mode with the appropriate filters.

Growth kinetics of the ΔXTFT *N. benthamiana* cell suspension cultures were measured in a 1 L flask containing 200 mL of cell culture. Triplicate samples were taken every 2 days. The dry weight of cells was determined by filtering a 2 mL sample through a pre-weighed 0.22 μm filter, then drying the filter and cells at 65 °C. Glucose and sucrose concentrations in the cell culture medium were measured using a YSI 2900 Biochemistry Analyzer (YSI Inc., Yellow Springs, OH, USA). Media samples were stored at −20 °C if not analyzed on the day of sampling. Until sucrose and glucose were depleted, media samples were diluted two-fold with distilled water prior to analysis.

### 4.4. Co-Cultivation of ΔXTFT N. benthamiana and Agrobacterium tumefaciens in Suspension Culture

For the mass ratio and media reduction co-culture experiments, the *Agrobacterium* vector used delivered a portion of the human CMG2 gene fused to a chimeric human IgG1/IgG2 Fc region with a secretion signal peptide (from the 2S albumin storage protein of *Arabidopsis thaliana*) at the 5′ end and the peptide sequence SEKDEL added to the 3′ end for ER retention [37]. *Agrobacterium* cultures were initiated by adding a 1% *v*/*v* inoculum of frozen (−80 °C) stock to LB media containing the appropriate antibiotics. After approximately 24 h, a 1% *v*/*v* inoculum from this initial culture was added to LB media containing antibiotics, 20 mM MES and 50 μM acetosyringone. After overnight growth, the *Agrobacterium* was centrifuged at 3000× *g* until supernatant was no longer cloudy (up to 30 min) and resuspended to the desired OD_600_ in sterile distilled water with 10 mM MgCl_2_, 20 mM MES and 150 μM acetosyringone. After a 3-hour incubation period at room temperature, the resuspended *Agrobacterium* solution was added to 7-day old plant cell cultures in 250 mL flasks with 50 mL culture. To initiate co-culture with different mass ratios of *Agrobacterium* to plant cells, equal volumes (1.5 mL) of *Agrobacterium* resuspended to different OD_600_ values were added each flask (containing 48 mL of plant cell culture). Dry cell weight of *Agrobacterium* was estimated using the following correlation: g dry weight/L = 0.6051 × OD_600_ [38]. In the media reduction experiment, plant cells (48 mL) were centrifuged for 5 min at 210*× g*, then a portion of the supernatant was removed prior to *Agrobacterium* addition (1.1 mL). After initiating co-culture, the shake flasks were incubated at 20 °C in the dark. The rotation rate of the shaker was 75 rpm for the first 18 h and was then increased to 140 rpm for the remainder of the experiment.

For the purification and glycoform characterization of CMG2-Fc, the *Agrobacterium* vector used delivered a portion of the human CMG2 gene fused to the human IgG1 Fc domain and targeted the protein for secretion using the rice alpha amylase 3D protein (RAmy3D) signal peptide without an ER retention signal [20]. Material was pooled from co-cultures in two 500 mL flasks (containing 98 mL plant cell culture and 8 mL *Agrobacterium*), one 1 L flask (containing 198 mL plant cell culture and 16 mL *Agrobacterium*) and four 2 L flasks (containing 398 mL plant cell culture and 32 mL *Agrobacterium*). Co-culture was performed as described above except antibiotics and additives were not used on the second day of *Agrobacterium* culture, 10 mM MES was used in the resuspension buffer, the *Agrobacterium* was washed twice in resuspension buffer prior to incubation and the resuspended *Agrobacterium* was incubated for 3 h at 20 °C on a rocking platform.

### 4.5. Extraction of CMG2-Fc for Co-Culture Parameter Experiments

Both cell-associated and extracellular CMG2-Fc were measured after 7 days of co-culture. Samples were centrifuged at 3000× *g* and 4 °C for 30 min to separate media (used to assess extracellular CMG2-Fc concentration) and cells. Extraction buffer (PBS containing 1 mM ethylenediaminetetraacetic acid (EDTA) and 2 mM sodium metabisulfite) was added to cells in a ratio of 1 mL buffer to 1 g fresh weight biomass. Samples were homogenized using a Tissue Tearor (BioSpec Products, Bartlesville, OK, USA) for 1 minute at full speed. After incubating for 10 min on ice, samples were centrifuged at 3000× *g* and 4 °C for 10 min to remove cell debris. Supernatant (cell-associated) and media (extracellular) samples were stored at −20 °C until analysis.

### 4.6. Purification of CMG2-Fc

After 7 days of co-culture, the cells were harvested for purification. The culture was centrifuged for 30 min at 3000× *g* and 4 °C to separate media and cells. Extraction buffer (PBS containing 1 mM EDTA and 2 mM sodium metabisulfite) was added to cells in a ratio of 1 mL buffer to 1 g fresh weight biomass. Cells in extraction buffer were stored overnight at 4 °C, then centrifuged for 10 min at 3000× *g* and 4 °C to separate cells from extraction buffer. Cells were homogenized in several batches (each consisting of ~150 g fresh weight biomass, ~2 g diatomaceous earth and 30 mL extraction buffer) using a Grindomix GM200 (Retsch, Haan, Germany) for 2 min at 5000 rpm. The remaining extraction buffer was added to the cells after homogenization. After mixing for 1 h at 4 °C, the homogenized cells were centrifuged for 1 h at 3000× *g* and 4 °C. The supernatant was filtered through a 0.45 and 0.2 μm capsule filter. Tangential flow filtration using a 30 kDa membrane was performed to concentrate the sample. Affinity chromatography was performed using 3 mL of MabSelect SuRe™ Protein A resin (GE Healthcare, Pittsburgh, PA, USA). Using a flowrate of 2 mL/min, 5 column volumes of PBS were used for equilibration. After sample loading, 15 column volumes of PBS were used to wash the column. For elution, 100 mM glycine, pH 2.5 was used and neutralized with 0.5 M Tris. Elution fractions were stored at −20 °C until analysis. Prior to N-glycosylation analysis, Amicon^®^ Ultra 0.5 mL centrifugal filter units with a 3 kDa membrane (MilliporeSigma, Burlington, MA, USA) were used for sample concentration and buffer exchange to 50 mM ammonium bicarbonate.

### 4.7. Quantification of CMG2-Fc

CMG2-Fc concentration was measured using a sandwich ELISA. Protein A (SouthernBiotech, Birmingham, AL, USA) was coated onto high-binding 96-well plates at a concentration of 50 μg/mL in PBS for 45 min at 37 °C. After blocking with 5% *w*/*v* nonfat dry milk in PBS for 15 min at 37 °C, standards and samples were added to the plate and incubated for 60 min at 37 °C. A standard curve was generated using pure, aglycosylated CMG2-Fc (supplied by Planet Biotechnology, Inc., Hayward, CA, USA). A goat anti-human IgG horseradish peroxidase conjugate (SouthernBiotech, Birmingham, AL, USA) was used for detection at a 1:2000 dilution and incubated for 45 min at 37 °C. SureBlue™ TMB substrate (KPL, Gaithersburg, MD, USA) was incubated at room temperature until color change was observed in standards. The reaction was stopped by adding 1 M HCl. The plate was washed three times for 10 min on a platform shaker with PBS containing 0.05% *v*/*v* Tween 20 before blocking, adding samples, adding detection antibody or adding TMB substrate. Absorbance at 450 nm was determined using a SpectraMax M2 microplate reader (Molecular Devices, Sunnyvale, CA, USA). Standards and samples were assayed in duplicate. Any readings outside the assay range were not included in subsequent analysis.

### 4.8. Gel Electrophoresis and Immunoblotting

SDS-PAGE was run using a 12% Mini-PROTEAN^®^ TGX™ precast gel (Bio-Rad Laboratories, Inc., Hercules, CA, USA). For immunoblotting, a goat anti-human IgG peroxidase conjugate (Sigma-Aldrich, St. Louis, MO, USA) was used as a detection antibody at a 1:10,000 dilution. TMB Stabilized Substrate (Promega, Madison, WI, USA) was used for color development. Samples were concentrated using Amicon^®^ Ultra 0.5 mL centrifugal filter units with a 10 kDa membrane (MilliporeSigma, Burlington, MA, USA). Prior to concentration, samples were filtered using a 0.2 μm syringe filter (MilliporeSigma, Burlington, MA, USA).

### 4.9. Site Specific N-linked Glycosylation Analysis

For N-linked glycopeptide analysis, samples were digested with trypsin to obtain glycopeptides. Before trypsin digestion, the samples were first reduced with 2 µL of 550 mM dithiothreitol (DTT) at 65 °C for 50 min in 50 mM ammonium bicarbonate (NH_4_HCO_3_) solution. Then 4 µL of 450 mM iodine acetamide (IAA) was used to alkylate samples for 25 min in the dark to prevent the re-formation of disulfide bonds between cysteines. One µg of trypsin was added to digest samples in a 37 °C water bath for 18 h overnight. To stop the digestion, samples were stored at −20 °C for 1 h. The instrument used was an Agilent 1290 infinity ultra-high-pressure liquid chromatography (UHPLC) system coupled to an Agilent 6495 triple quadrupole (QQQ) mass spectrometer. Solvents of the 10 min LC gradient include solvent A of 3% acetonitrile and solvent B of 90% acetonitrile in nanopore water. Samples were first separated with an Agilent Eclipse plus C18 column (RRHD 1.8 µm, 2.1 × 150 mm) connected to an Agilent Eclipse plus C18 trap column (RRHD 1.8 µm, 2.1 × 5 mm). The tandem mass spectrometry mode operated on the instrument was dynamic multiple reaction monitoring (MRM). Data collected from the instrument were analyzed with the Agilent MassHunter Quantitative Analysis software (B.05.02, Agilent, Santa Clara, CA, USA).

## 5. Conclusions

An anthrax decoy fusion protein was successfully produced using *Agrobacterium*-mediated transient expression in ΔXTFT *N. benthamiana* cell culture by simply adding recombinant *Agrobacterium* to the cell suspension culture and harvesting the biomass and cell culture broth after 7 days of co-cultivation. Reduced levels of plant-specific N-glycans were observed compared to transient production of the same protein in wild type *N. benthamiana* plants. The N-glycan distribution from this production system could be further tuned by utilizing knockout transgenic cell lines that can be generated using CRISPR/Cas9 [33,34] or generating cell cultures from ΔXTFT *N. benthamiana* plants that also express the mammalian sialylation pathway [35]. A key advantage of this transient expression system is that the *Agrobacterium* constructs required to produce different recombinant proteins can be quickly and easily made. Additionally, bioreactor-based plant transient expression would enable environmental control and optimization and could simplify purification by targeting the protein for secretion. Though additional process development is needed to enhance expression levels, this method is a potential scalable platform technology for rapid production of novel protein therapeutics.

## Figures and Tables

**Table 1:** Growth kinetic parameters for ΔXTFT *N. benthamiana* shake flask cultures.

Initial Biomass Concentration ^1^ (g Dry Weight/L)	Maximum Biomass Concentration ^1^ (g Dry Weight/L)	Maximum Specific Growth Rate ^2^ (day^−1^)	Yield Coefficient ^2^ (g Dry Weight Biomass/g glucose)
0.9 ± 0.2	7.4 ± 0.5	0.113 ± 0.004	0.55 ± 0.04

^1^ Average ± standard deviation of triplicate samples; ^2^ Value ± standard error for linear regression coefficient.
